# Urban Mind: Using Smartphone Technologies to Investigate the Impact of Nature on Mental Well-Being in Real Time

**DOI:** 10.1093/biosci/bix149

**Published:** 2018-01-10

**Authors:** Ioannis Bakolis, Ryan Hammoud, Michael Smythe, Johanna Gibbons, Neil Davidson, Stefania Tognin, Andrea Mechelli

**Affiliations:** 1 Reader, PhD student at the Institute of Psychiatry, Psychology and Neuroscience at King's College London, United Kingdom; 2 Lecturer, PhD student at the Institute of Psychiatry, Psychology and Neuroscience at King's College London, United Kingdom; 3 Lecturer, PhD student at the Institute of Psychiatry, Psychology and Neuroscience at King's College London, United Kingdom; 4 PhD student at the Institute of Psychiatry, Psychology and Neuroscience at King's College London, United Kingdom; 5 Artist and action-based researcher at Nomad Projects (*https://nomadprojects.org*) in London, United Kingdom; 6 Landscape architects at J & L Gibbons (*http://jlg-london.com*) in London, United Kingdom

**Keywords:** nature, mental well-being, mental health, smartphones, ecological momentary assessment

## Abstract

Existing evidence on the beneficial effects of nature on mental health comes from studies using cross-sectional designs. We developed a smartphone-based tool (Urban Mind; *www.urbanmind.info*) to examine how exposure to natural features within the built environment affects mental well-being in real time. The tool was used to monitor 108 individuals who completed 3013 assessments over a 1-week period. Significant immediate and lagged associations with mental well-being were found for several natural features. These associations were stronger in people with higher trait impulsivity, a psychological measure of one's tendency to behave with little forethought or consideration of the consequences, which is indicative of a higher risk of developing mental-health issues. Our investigation suggests that the benefits of nature on mental well-being are time-lasting and interact with an individual's vulnerability to mental illness. These findings have potential implications from the perspectives of global mental health as well as urban planning and design.

**Over three-and-a-half billion people, more than half** the world's population, live in urban areas. This number is rising fast in both developed and developing countries, and it is expected that 66% of the global population will live in cities by 2050 (UNESA 2014). This ongoing urbanization has major implications for global mental health, because people who live in urban environments are at higher risk of a range of mental-health issues, including depression, generalized anxiety disorders, psychosis, and addictive disorders (Peen et al. 2010, Galea 2011, Lederbogen et al. 2011). Crucially, the observation of a dose-dependent effect provides support for a causal relationship, rather than a mere association, between urban living and risk for mental illness (Pedersen and Mortensen 2001, Haddad et al. 2015). Further evidence for this causal relationship comes from the finding that the adverse impact of urban living can be reversible, with some categories of patients showing improved clinical outcomes after moving from urban to rural environments (Pedersen and Mortensen 2001). Although city dwellers are at higher risk of mental illness, an increasing body of evidence suggests that natural features within the built environment can counteract the detrimental effects of urban living and even promote mental health. For example, living in urban areas with natural features such as trees, gardens, parks, birds, and water is associated with higher levels of mental well-being and reduced incidence of chronic mental illness (van den Berg et al. 2010, van Dillen et al. 2012, Astell-Burt et al. 2013, Nutsford et al. 2013, Richardson et al. 2013, White et al. 2013, Astell-Burt et al. 2014, Alcock et al. 2015, Mantler and Logan 2015, Taylor et al. 2015, Triguero-Mas et al. 2015, Cox et al. 2017). A number of biologically plausible theories have been proposed to explain this effect, including attention-restoration theory (Kaplan S 1995), stress-reduction theory (Ulrich et al. 1991), and biophilia theory (Wilson 1993).

The existing literature on the beneficial impact of nature on mental health, however, is hindered by a number of limitations. First, most studies have used a cross-sectional design involving the acquisition of a single “snapshot,” without accounting for the fact that people experience a diverse range of urban environments throughout the day. Second, most research has examined the role of green spaces per se without considering the type and amount of nature people require to experience beneficial effects on their mental health. Third, most studies have attempted to measure the impact of green spaces without assessing how this might depend on individual characteristics such as age, gender, lifestyle, and mental health. For example, the beneficial effects of nature might be particularly evident in those individuals who, due to pre-existing genetic and environmental factors, possess greater vulnerability to mental-health issues. In light of these limitations, a better understanding of how natural features of the urban environment affect mental health remains an urgent priority, as was highlighted by the World Health Organization in their declaration of 2010 as the Year of Urban Health (WHO and UN-Habitat 2010).

## Overcoming the limitations of the existing literature with smartphone-based ecological momentary assessment

In order to overcome the limitations of the existing literature, we have developed a smartphone app for examining the impact of the surrounding built environment on mental well-being (a strong predictor of mental health) as people go about their daily lives. The Urban Mind app uses a methodology known as *ecological momentary assessment*, which involves repeated sampling of current experiences in real-time and real-world contexts (Shiffman et al. 2008). The use of smartphone-based ecological momentary assessment has three significant benefits: first, it allows multiple measurements over time, providing insight into dynamic changes in mental states that could not be captured by a single snapshot; second, it allows the acquisition of detailed information on the type and amount of nature that people experience either incidentally or intentionally; third, it maximizes ecological validity as data are collected in real-world environments (Beute et al. 2016). A further advantage of using smartphones to collect research data is that people tend to carry and use them multiple times as part of their daily lives; in contrast, the deployment of paper diaries or stand-alone electronic devices places greater demands on the individual, resulting in high rates of missing responses. The use of smartphones is also cost-effective: Once the expense of developing an app has been met, it can easily be downloaded and installed on any smartphone worldwide, allowing large numbers of participants to provide research data with minimal operational costs.

## The Urban Mind tool

With our technical provider Artists & Engineers (*www.artistsandengineers.co.uk*), we developed a native software application (app) named Urban Mind using the Ionic cross-platform development framework, compatible with any iPhone devices running under iOS 7.1 or newer and any smartphone running under Android 4.4 or newer. We also developed back-end server software to communicate with the app and a public-facing, project-related website providing information on the research to potential participants (*www.urbanmind.info*). The Urban Mind tool could be downloaded for free from the Apple App Store and the Google Play Store. The participants were recruited over a period of 8 months (October 2015–June 2016) using the project-related website and social-media platforms. In particular, an Urban Mind page was created on Instagram (*www.instagram.com/urban_mind_project*), Twitter (*www.twitter.com/Urban_Mind_Proj*), and Facebook (*www.facebook.com/UrbanMindProject*) to disseminate the project and encourage participation. In addition, recruitment of the participants was enhanced by coverage of the project on mainstream national and international media. Participation in the project was self-selected and anonymous. After downloading and installing the app on their smartphones, the participants were presented with a description of the aim and methodology of the project and were asked to provide informed consent. Once informed consent was granted, the app acquired the following data:

-  A baseline assessment of demographics (e.g., age, gender, and ethnicity), socioeconomics (e.g., education and occupation), mental well-being, and trait impulsivity. Mental well-being was assessed using the Warwick-Edinburgh Mental Well-Being Scale, which focuses on the previous 2 weeks (Tennant et al. 2007); a full list of questions for this scale and a description of how they were scored can be found in the supplemental material (see supplemental scale S1). Trait impulsivity was measured using the Trait Rash Impulsivity Scale (Mayhew and Powell 2014); a full list of questions and a description of how these were coded is available in the supplemental material (see supplemental scale S2).
-  A total of 49 ecological momentary assessments, with 7 per day over a period of 7 days. Each ecological momentary assessment lasted about 2 minutes and covered the following three areas: (1) an individual's perception of their surrounding environment, assessed through a series of questions tailored to whether the participant was indoors or outdoors; (2) an individual's geographical location using GPS-based geotagging; and (3) an individual's momentary mental well-being. Momentary mental well-being was assessed via an adapted version of the Warwick-Edinburgh Mental Well-Being Scale, which focused on the present moment (Tennant et al. 2007); a full list of questions for this scale and a description of how they were scored can be found in the supplemental,material (see supplemental scale S3). For the purpose of the present investigation, we focus on six questions of interest that refer to natural features of the surrounding environment: Are you indoors or outdoors? Can you see trees? Can you see the sky? Can you hear birds singing? Can you see or hear water? Do you feel in contact with nature? Possible answers to each question included *yes, no*, and *not sure*.

When people received a prompt to complete an ecological momentary assessment, they had 30 minutes to do so before this was marked as incomplete. This allowed users a time frame to respond to the prompt without the need to interrupt any activities they were engaged in. If the Internet was inaccessible at the time of an ecological momentary assessment, the data were stored on the device and uploaded when mobile or Wi-Fi Internet access was next available.

**Figure 1.**
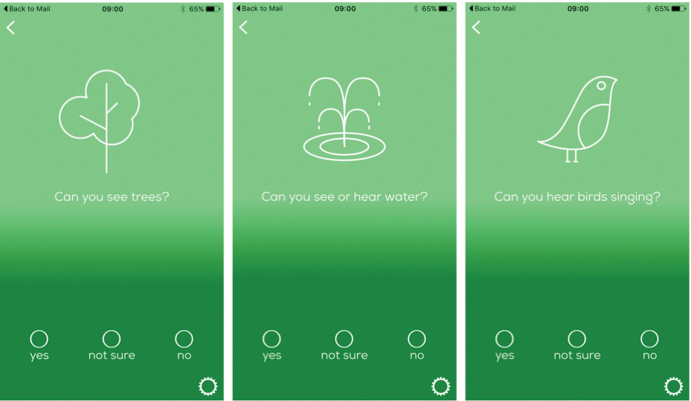
Screenshots of the Urban Mind tool.

Within each ecological momentary assessment, different questions were associated with distinct icons (see figure 1 for examples). This extensive use of iconography had two advantages: first, it made the experience of using the tool more enjoyable, thereby promoting sustained engagement; second, it shortened the time required to complete an ecological momentary assessment, because users learned to recognize each question from the associated icon.

**Figure 2.**
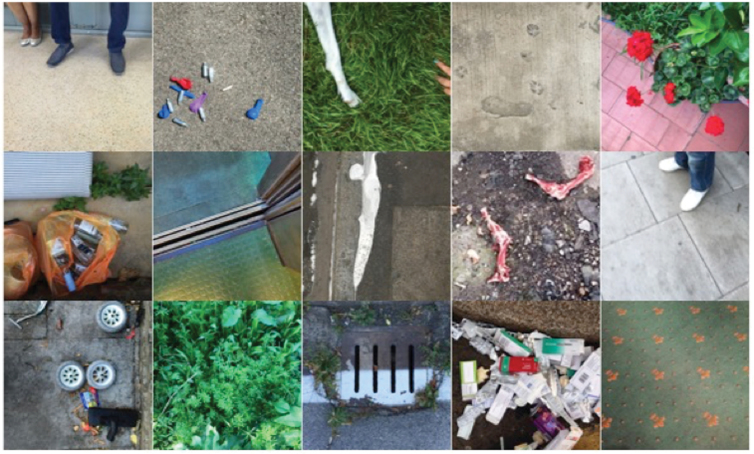
Selection of photographs submitted by participants using the Urban Mind tool.

Each time a participant completed an ecological momentary assessment, they were invited to collect and submit a photograph of the ground and/or an audio recording of the surrounding environment (see figure 2 for a selection of the photographs). The rationale for inviting participants to document their surrounding environment was twofold: first, these photographic and audio recordings were used to disseminate the research on the project-related website and social media platforms; second, the activity of documenting one's environment was meant to promote sustained engagement with the tool as well as the wider research project. In addition, we envisage that these photographic and audio recordings could be treated as research data amenable to scientific investigation in the future; for example, GIS-based time-graphic methods could be applied to the photographic and audio recordings to generate a geonarrative account of the data (Kwan and Ding 2008).

## Using the Urban Mind tool to investigate the impact of nature on mental well-being in real time

We used the Urban Mind app to monitor exposure to natural features of the built environment and associated changes in mental well-being in 108 participants for a period of 1 week. One of the aims of our project was to explore how the impact of the surrounding built environment on the mental well-being of an individual might depend on their vulnerability to developing mental-health issues. For this reason, we included an assessment of trait impulsivity, a well-established psychological measure of one's tendency to behave with little forethought, reflection, or consideration of the consequences (Mayhew and Powell 2014). Animal and human models suggest that this measure is predictive of future risk of developing mental-health issues including addictive disorders (McChargue et al. 2011, Kunst and Van Wilsem 2013, Velázquez-Sánchez et al. 2014, Sanchez-Roige et al. 2014, Dalley and Robbins 2017), attention-deficit hyperactivity disorder (Ahmad and Hinshaw 2017, Martel et al. 2017), antisocial personality disorder (González et al. 2016, Martel et al. 2017), and bipolar disorder (Tu et al. 2017). We used the data to test three related hypotheses: (1) exposure to natural features, including trees, the sky, birdsong, and water, would be associated with higher levels of momentary mental well-being; (2) these effects would still be evident in subsequent assessments, in which people are no longer exposed to trees, the sky, birdsong, and water, indicating time-lasting benefits; (3) these effects would be more evident in people with higher trait impulsivity, who are thought to carry greater vulnerability to mental-health issues, than those with lower trait impulsivity (Dalley and Robbins 2017).

The research project had received full ethical approval from the Psychiatry, Nursing, and Midwifery Research Ethics Sub-Committee at King's College London (no. PNM/14/15-59). The data generated during the current project are available from the corresponding author on request.

### Statistical analysis

The statistical analysis of the data was performed using STATA 14.1. Our main analysis was based on 2094 assessments from 64 participants who completed a minimum of 25 assessments each (more than a 50% response rate). We also ran two additional analyses, including 108 participants with a response rate of 33% (total number of assessments: 3013) and 25 participants with a response rate of 66% (total number of assessments: 966).

Longitudinal associations between self-reported environmental features of interest and momentary mental well-being were investigated using random intercept linear models and expressed as mean differences of momentary mental well-being. All models were adjusted for the following potential confounders: gender, age, occupational status, and mental well-being over the 2 previous weeks. In practice, this adjustment was implemented by including these variables as additional covariates within each statistical model. The interaction effect between self-reported environmental features and participant trait impulsivity on mental well-being was assessed by including an interaction term in the random intercept linear models. For more information on the implementation of the statistical analysis, see “Technical Note” in the supplemental material.

In addition, to explore the association between self-reported environmental features and momentary mental well-being at specific and concurrent time points, we created time-shifted copies of the self-reported environmental variables and included them in the random intercept regression models. The relationships of all the explanatory variables may be immediate or occur with some lag; therefore, we examined models that included multiple lags of the explanatory variables. Because the interval between successive ecological momentary assessments was 2 hours and 25 minutes on average, one-lagged effects would indicate that the impact of a feature of interest was still evident after 2 hours and 25 minutes, two-lagged effects would indicate that the impact of a feature of interest was still evident after 4 hours and 50 minutes, and three-lagged effects would indicate that the impact of a feature of interest was still evident after 7 hours and 15 minutes. Because multiple lags could be correlated with each other, we also allowed the effect of environmental variables to decline with time with the use of second-order polynomial-distributed lag models.

Finally, we repeated our statistical analyses using the STATA routine ice, an implementation in STATA of the Multiple Imputations with Chained Equations (MICE) procedure, and compared our results with the original analysis under the missing at random (MAR) assumption (White and Royston 2009).

All statistical models were tested for multicollinearity using Pearson and Goodman and Kruskal's gamma correlation coefficients; this indicated that multicollinearity was not an issue, as we show in supplemental table S1.

### The demographic characteristics of the participants

The demographic characteristics of the participants are presented in table 1. In brief, our sample of 108 participants included 30 males and 78 females with an average age of 31.1 years (age range: 20–67). Over 91% of the participants were educated to the university level, and the vast majority of them were either studying (32.4%) or working (45.4%). All 108 participants completed at least 33% of the assessments; 64 participants completed at least 50%, and 25 participants completed at least 66%. It can be seen from table 1 that those completing a higher number of assessments were more likely to be a student when compared with those completing a lower number of assessments, who were more likely to be working. In contrast age, gender, and mental well-being over the previous 2 weeks appeared to be comparable between high and low responders (table 1).

**Table 1.** Descriptive statistics of the sample of participants for the different percentages of assessments (33%, 50%, 66%) being completed. The percentages are calculated for the number of participants and the number of responses.

	Number (%) unless otherwise stated	Number (%) unless otherwise stated	Number (%) unless otherwise stated
Number of participants	*n* = 25 (66%)	*n* = 64(50%)	*n* = 108 (33%)
Male	6 (25%)	15 (23%)	30 (27%)
Age	Mean: 31.4 SD: 12.26	Mean: 30.8 SD: 11.8	Mean: 31.1 SD: 11.1
Occupational status			
Employed	7 (28.0%)	27 (42.2%)	49 (45.4%)
Retired	1 (4.0%)	2 (3.1%)	2 (1.9%)
Self-employed	3 (12.0%)	6 (9.4%)	14 (13.0%)
Student	12 (48.0%)	23 (35.9%)	35 (32.4%)
Unemployed	2 (8.0%)	6 (9.4%)	8 (7.4%)
Level of education			
University	24 (96.0%)	60 (93.7%)	99 (91.7%)
Training college	1 (4.0%)	1 (1.6%)	4 (3.7%)
Secondary school	0 (0.0%)	2 (3.1%)	4 (3.7%)
Apprenticeship	0 (0.0%)	1 (1.6%)	1 (0.9%)
Mental-well-being score over previous 2 weeks	Mean: 47.4 SD: 9.6	Mean: 48.7 SD: 8.8	Mean: 48.3 SD: 9.6
Momentary mental well-being score	Mean: 52.5 SD: 12.8	Mean: 53.2 SD: 11.8	Mean: 52.7 SD: 12.0
Trait-impulsivity score over previous 2 weeks	Mean: 9.6 SD: 4.0	Mean: 9.8 SD: 3.7	Mean: 10.1 SD: 3.9
Number of assessments	Mean: 39.0 SD: 4.1	Mean: 33.7 SD: 5.8	Mean: 29.9 SD: 7.5
Are you indoors or outdoors?			
Indoors	832 (88.4%)	1765 (86.9%)	2483 (85.5%)
Outdoors	109 (11.6%)	265 (13.1%)	422 (14.5%)
Can you see trees?			
Yes	570 (60.6%)	1203 (59.3%)	1802 (62.0%)
No/Not Sure	371 (39.4%)	827 (40.7%)	1103 (38.0%)
Can you hear birds singing?			
Yes	49 (44.6%)	115 (43.2%)	192 (45.3%)
No or not sure	61 (55.4%)	151 (56.8%)	232 (54.7%)
Can you see or hear water?			
Yes	25 (22.7%)	56 (21.1%)	95 (22.4%)
No or not sure	85 (77.3%)	210 (78.9%)	329 (77.6%)
Can you see the sky?			
Yes	598 (71.8%)	1210 (68.5%)	1746 (70.3%)
No or not sure	235 (28.2%)	556 (31.5%)	738 (29.7%)
Do you feel in contact with nature?			
Yes	67 (61.5%)	152 (57.4%)	246 (58.3%)
No or not sure	42 (38.5%)	113 (42.6%)	176 (41.7%)

*Abbreviations:* SD, standard deviation

### The association between natural features and mental well-being

The statistical analysis adjusted for mental well-being over the previous 2 weeks showed positive associations between momentary mental well-being and being outdoors (*p* < .001), seeing trees (*p* < .001), hearing birds singing (*p* < .001), seeing the sky (*p* < .001), and feeling in contact with nature (*p* < .001) across the three different response-rate thresholds (33%, 50%, and 66%). In contrast, an association between momentary mental well-being and being able to see or hear water was only observed at the 66% response-rate threshold. For most features of interest, effect estimates did not differ significantly when using the MICE procedure (table 2).

**Table 2.** Associations between momentary mental well-being score and self-reported environmental features adjusted for potential confounders defined a priori.

	Model 1^a^ (*n* = 108)		Model 1^a^ (*n* = 64)		Model 1^a^ (*n* = 25)		Model 2^b^ (*n* = 64)		Model 3^c^ (*n* = 64)	
	MD	95% CI	MD	95% CI	MD	95% CI	MD	95% CI	MD	95% CI
Are you indoors or outdoors?	**2.30**	**1.51–3.09**	**2.40**	**1.42–3.37**	**2.89**	**1.46–4.31**	**2.42**	**1.36–3.47**	**2.44**	**1.38–3.49**
Can you see trees?	**1.77**	**1.13–2.41**	**1.81**	**1.05–2.57**	**1.31**	**0.16–2.45**	**1.80**	**1.05–2.56**	**1.81**	**1.05–2.57**
Can you hear birds signing?	**3.84**	**2.23–5.45**	**3.81**	**1.86–5.76**	2.49	–0.26–5.25	**3.89**	**1.61–6.17**	**3.95**	**1.56–6.34**
Can you see or hear water?	1.31	–0.57–3.19	1.27	–1.00–3.54	**3.23**	**0.17–6.33**	1.54	–2.00–5.07	1.51	–2.04–5.06
Can you see the sky?	**1.41**	**0.69–2.14**	**1.62**	**0.79–2.46**	0.95	0.20–2.19	**1.48**	**0.47–2.50**	**1.49**	**0.47–2.50**
Do you feel in contact with nature?	**3.74**	**2.17–5.31**	**3.51**	**1.61–5.40**	1.27	–1.30–3.84	**3.71**	**1.92–5.51**	**3.72**	**1.90–5.55**

*Note:* Mean difference (MD) and 95% confidence intervals (CI) represent a mean difference in momentary mental well-being score per category increase (compared with the reference group). Statistically significant effects (p < .05) are highlighted in bold.

a Controlled for mental well-being over the previous 2 weeks.

b Controlled for mental well-being over the previous 2 weeks, plus the Multiple Imputation with Chained Equations (MICE) procedure was employed for the participants who completed more than 50% of the momentary assessments.

c Controlled for age, gender, occupational status, and mental well-being over the previous 2 weeks, plus MICE procedure was employed for the participants who completed more than 50% of the momentary assessments.

**Figure 3.**
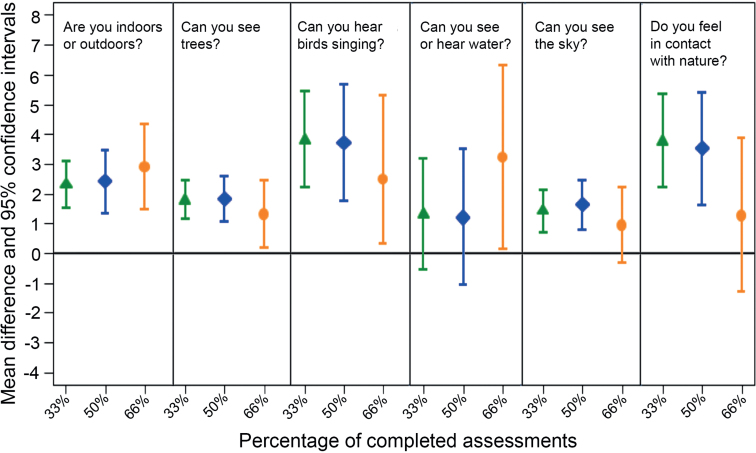
Associations between momentary mental well-being score in relation to self-reported environmental features adjusted for age, gender, occupational status and mental well-being score over the previous two weeks for different thresholds of completed assessments (>33%, >50%, and >66%). The mean difference (MD) refers to the mean difference in momentary mental well-being score per category increase. The horizontal axis represents the different thresholds from 33%, 50%, and 66%, and the vertical axis represents the MD with 95% confidence interval. See supplemental Table S2 for more details.

These results were replicated when, in addition to adjusting for mental well-being over the previous 2 weeks, we further adjusted for gender, age, and occupational status. Here, consistent associations with momentary mental well-being across the three different response-rate thresholds (33%, 50%, and 66%) were seen for being outdoors (*p* < .001) and seeing trees (*p* < .001). Associations with momentary mental well-being across two thresholds (33% and 50%) were found for hearing birds singing (*p* < .001) and feeling in contact with nature (*p* < .001). Specifically, the effect sizes for being outdoors versus being indoors were 2.31 (95% confidence intervals [CI] = 1.52–3.11), 2.41 (95% CI = 1.43–3.39), and 2.90 (95% CI = 1.48–4.33) for the 33%, 50%, and 66% thresholds, respectively; the effect sizes for seeing trees versus not seeing trees were 1.78 (95% CI = 1.14–2.42), 1.82 (95% CI = 1.06–2.57), and 1.31 (95% CI = 0.16-2.46), respectively. The effect sizes for hearing birds singing versus not hearing birds singing, across the 33% and 50% thresholds, were 3.82 (95% CI = 2.21–5.43) and 3.71 (95% CI = 1.75–5.68), respectively; the effect sizes for feeling in contact with nature, across the 33% and 50% thresholds, were 3.77 (95% CI = 2.20–5.34) and 3.51 (95% CI = 1.62–5.41), respectively (see figure 3 and supplemental table S2).

### The time-lag association between natural features and mental well-being

**Figure 4.**
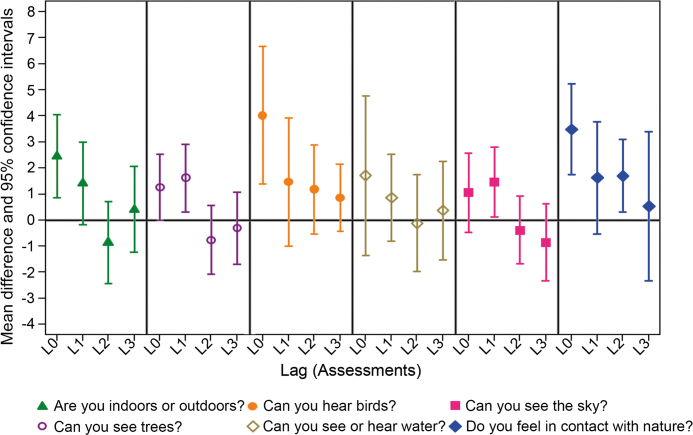
Associations of momentary mental well-being score in relation to self-reported environmental characteristics adjusted for age, gender, occupational status, and mental well-being over the previous two weeks with the use of the MICE procedure. The mean difference (MD) refers to the mean difference in momentary mental well-being score per category increase. The horizontal axis represents the assessments lagged from 0–3, and the vertical axis represents the MD with 95% confidence interval. L0 indicates the impact of a feature of interest on momentary well-being at the time of the first assessment; L1 indicates that the impact of a self-reported environmental feature of interest was still evident after 2 hours and 25 minutes; L2 would indicate that the impact of a feature of interest was still evident after 4 hours and 50 minutes; and L3 would indicate that the impact of a feature of interest was still evident after 7 hours and 15 minutes. See supplemental Table S3 for more details.

The use of models with multiple lags revealed a number of significant lagged effects, indicating a time-lasting effect of nature on momentary mental well-being, after adjusting for gender, age, occupational status, and mental well-being over the previous 2 weeks. In particular, seeing trees and seeing the sky during an assessment had a statistically significant effect on momentary well-being in the next subsequent assessment, which took place an average of 2 hours and 25 minutes later (*p* values: 0.015 and 0.031, respectively). The effect size of seeing trees on momentary mental well-being in the next subsequent assessment was 1.62 (95% CI = 0.31–2.92), whereas the effect size of seeing the sky on momentary mental well-being in the next subsequent assessment was 1.46 (95% CI = 0.13–2.80). In addition, feeling in contact with nature during an assessment had a statistically significant effect on momentary mental well-being during the second subsequent assessment, which took place an average of 4 hours and 50 minutes later (*p* value: .018), with an effect size of 1.70 (95% CI = 0.31–3.10). The associations between the six different environmental features under investigation and momentary mental well-being with different lag effects are shown in figure 4 and supplemental table S3.

### The interaction between natural features and trait impulsivity on mental well-being

**Figure 5.**
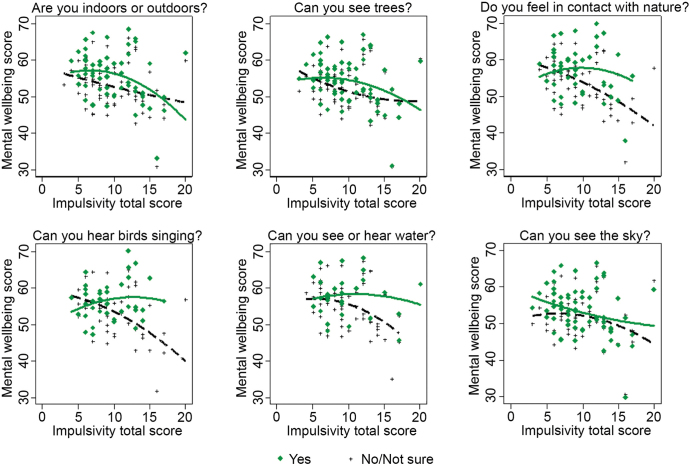
Mental well-being total score versus trait impulsivity score for participants with completed assessments > 50% with the use of random intercept linear models. Group-specific quadratic regression curves are shown for those that responded Yes and for those that responded No/Not sure to the questions “Are you indoors or outdoors?”, “Can you see trees?”, “Do you feel in contact with nature?”, “Can you hear birds singing?”, “Can you see or hear water?”, and “Can you see the sky?”. P-value for interaction term for the question “can you see the sky?” > 0.05. All the other p-values for the interaction terms < 0.05. See supplemental Table S4 for more details.

There was a statistically significant interaction between exposure to our environmental features of interest and trait impulsivity on momentary mental well-being. In particular, the effects of being outdoors (*p* value for interaction term: .037), seeing trees (*p* value for interaction term: .026), hearing birdsong (*p* value for interaction term: .021), hearing birds singing (*p* value for interaction term: .001), and feeling in contact with nature (*p* value for interaction term: .025) were greater in people with higher levels of trait impulsivity than those with lower levels of trait impulsivity. Figure 5 and supplemental table S4 present the interaction terms for the six natural features under investigation.

## Cross-disciplinary implications of the findings

The aim of this study was to use a novel smartphone-based tool to monitor the impact of nature on momentary mental well-being in real-time and real-world environments. Consistent with our first hypothesis, we found that being outdoors, seeing trees, hearing birds singing, seeing the sky, and feeling in contact with nature were associated with higher levels of momentary mental well-being; in contrast, a consistent effect was not detected for seeing or hearing water. These findings provide empirical support for the notion that short-term exposure to specific natural features has measurable beneficial effects on mental well-being, complementing existing evidence from previous studies that examined cumulative long-term exposure to nature (Kaplan 2001, Evans 2003, Velarde et al. 2007, Grinde and Patil 2009, Sarkar et al. 2013, Cox et al. 2017). In addition, consistent with our second hypothesis, we found that these beneficial effects could still be observed even if the participant was no longer outdoors and no longer had access to nature. This lagged effect indicates a time-lasting impact of nature on mental well-being that can still be observed after several hours. Finally, consistent with our third hypothesis, we found that the effects of being outdoors, seeing trees, seeing the sky, hearing birds singing, and feeling in contact with nature on momentary mental well-being were greater in people with higher levels of impulsivity. In light of the associations between impulsivity and addictive disorders (McChargue et al. 2011, Kunst and Van Wilsem 2013, Sanchez-Roige et al. 2014, Velázquez-Sánchez et al. 2014, Dalley and Robbins 2017), attention-deficit hyperactivity disorder (Ahmad and Hinshaw 2017, Martel et al. 2017), antisocial personality disorder (González et al. 2016, Martel et al. 2017), and bipolar disorder (Tu et al. 2017), this finding supports the notion that the beneficial effects of nature may be especially evident in those individuals who possess greater vulnerability to mental-health issues.

The implications of these results are twofold. First, from the perspective of urban planning and design, the data provide a much-needed evidence base that could inform future investments and policies. There is an urgent need for such an evidence base because at present, decisions on urban planning and design aimed at improving the mental health of the general population tend to be based on “conventional wisdom” because of the lack of robust scientific data. In particular, our findings suggest that greater access to natural features within the built environment can be especially beneficial to individuals who show higher levels of impulsivity and, as such, may be at greater risk of developing mental-health issues. Second, from the perspective of mental health, the data could inform the development of low-cost scalable interventions aimed at promoting mental health in urban populations. This is an urgent global health priority because mental illness is the leading causes of disability worldwide, accounting for 32.4% of years lived with disability (YLDs) and 13% of disability-adjusted life years (DALYs; Vigo et al. 2016). In particular, our findings suggest that increasing and promoting access to nature could be a simple but effective intervention to help vulnerable individuals maintain high levels of mental well-being, thereby reducing risk of developing a mental illness. This conclusion extends a previous report of a beneficial effect of nature in patients with attention-deficit hyperactivity disorder to a nonclinical population (Kuo and Taylor 2004).

From a methodological perspective, our investigation shows that it is feasible to use smartphone-based ecological momentary assessment to examine the relationship between specific aspects of the built environment and mental well-being as people go about their daily life. A crucial advantage of this approach is that it maximizes ecological validity, because data are collected in real-world environments (Beute et al. 2016). A further advantage is that it minimizes recall bias—a common issue in psychological research—because data are collected in real time (Shiffman et al. 2008). In comparison with cross-sectional studies, our approach also allows the investigation of both immediate and lagged effects within individuals, allowing us to capture dynamic temporal associations that would be diluted in population-based surveys.

In addition, our investigation shows that it is feasible to use smartphone-based ecological momentary assessment to test for interactions between the environment and the individual. For example, through the inclusion of a well-established measure of trait impulsivity as part of the baseline assessment, we were able to demonstrate an interaction between natural features of the built environment and an individual's susceptibility to specific mental-health issues characterized by addictive and externalising behaviours. However, we know that *all* mental disorders are the results of the complex interplay between environmental and individual factors. Therefore, future studies could replace trait impulsivity with transdiagnostic measures of mental health (e.g., the Mental Health Continuum; Oprana et al. 2017).

### Limitations

Our investigation has a number of limitations. First, because the data were acquired using an observational rather than an experimental design, it was not possible to establish whether the observed associations between nature and mental well-being reflect a direct causal impact of the former on the latter. One could speculate, for example, that individuals who already feel low may be less likely to leave the home and experience natural habitat. However, the observation of time-lagged effects, in which the beneficial effects of nature could still be detected after the participant was no longer exposed to trees, the sky, and birdsong, provides indirect support to our interpretation that nature had a beneficial impact on mental well-being. Second, the present investigation relied on self-reports, which are known to be prone to potential bias (Shiffman 2014). For example, individuals with lower levels of mental health tend to pay greater attention to negative than positive stimuli (Mogg et al 1995); for example, someone with low mental well-being might have paid more attention to aversive traffic sounds than sounds of birds or water. Third, our sample comprised smartphone users with higher-than-average levels of education and an average age of just 31.1 years and therefore cannot be considered representative of the general population. In addition, this sample was not randomly derived from a specific population because it was based on participants choosing to download and use the app. However, it was not our aim to make inferences about a specific population, and because of the self-selected nature of the sample, we did not think it would be appropriate to calculate sampling weights. Future studies would benefit from recruiting a more diverse sample and investigating how the results change as a function of demographic and socioeconomic factors. This could be achieved, for example, through the use of “ambassadors” from a wide range of demographic and socioeconomic backgrounds who could disseminate the project and promote participation among their peers and communities. Finally, it is important to acknowledge that mental well-being and mental health are not the same: *mental well-being* refers to positive states of being, feeling, thinking, and behaving, whereas *mental health* incorporates a range of negative and positive states, from several mental illness to excellent mental health (Tennant et al. 2007). Nevertheless, mental well-being is thought to be good indicator of the extent to which individuals are able to function and thrive in their day-to-day life and is predictive of future risk of mental illness (Keyes 2007).

### Comparison with previous research

It should be noted that previous studies have employed smartphone-based ecological momentary assessment to examine the relationship between the built environment and mental well-being *in situ*. For example, MacKerron and Mourato (2013) developed an app that combines GPS information with mood ratings and reported that exposure to natural environments leads to greater “happiness.” Other studies employed smartphone technologies to measure mental well-being within a specific natural habitat of interest (Doherty et al. 2014) or evaluate the beneficial impact of a brief exposure to nature on cognition (Berman et al. 2012). Our investigation, however, provides a distinct contribution for several reasons. First, we assessed the impact of self-reported exposure to specific natural features of interest (e.g., “can you see trees?”) irrespective of whether these were located within a rural or an urban environment. In contrast, previous studies used GPS information to infer the habitat (e.g., rural versus urban) in which an individual was located at the time of each ecological momentary assessment and therefore were unable to capture the impact of natural features within the urban environment (MacKerron and Mourato 2013). Second, we estimated not only immediate but also lagged associations with momentary mental well-being; this revealed a time-lasting effect of exposure to nature on mental well-being that, to our knowledge, has not been reported in previous studies using the ecological momentary assessment technique. Finally, through the inclusion of a scale measuring trait impulsivity in the baseline assessment, we were able to demonstrate that the impact of natural features on mental well-being depends on an individual's personality. Despite these distinct aspects, the findings of our investigation are consistent with the results of these earlier studies, providing further empirical evidence to the notion that nature can have a beneficial impact on mental well-being.

## Conclusions

In conclusion, our investigation represents a successful example of citizen science, enhanced by the development of a project-related social media (e.g., Instagram, Twitter, and Facebook). Our findings indicate that (a) it is feasible to use smartphone-based ecological assessment to examine the relationship between natural features of the built environment and momentary mental well-being in real time; (b) exposure to natural features including trees, the sky, and birdsong has a time-lasting beneficial impact on momentary mental well-being; and (c) the beneficial effects of nature may be especially evident in those individuals with greater levels of trait impulsivity who are at greater risk of developing addictive disorders and other mental-health issues. These findings have potential implications from the perspectives of global mental health and urban planning and design.

## Supplementary Material

Supplementary Data
