# Supplementary material for: Urban Mind: Using Smartphone Technologies to Investigate the Impact of Nature on Mental Well-Being in Real Time
Source: Bioscience. 2018 Jan 10;68(2):134–45. doi: 10.1093/biosci/bix149 (PMC5862247; doi:10.1093/biosci/bix149)
Supplement: Supplementary Data [file bix149_supp.docx]

**SUPPLEMENTAL MATERIAL**

**Technical Note**

The study data represent a very large, unbalanced panel, with large N (the number of individuals) and highly variable T (the number of assessments per individual). Let $y_{it}$ be the total score of self–reported mental wellbeing of individual *i* for assessment *t*. We model these continuous data using a random intercept linear model with

$i=1,2, \ldots$ N

$t=1,2, \ldots T$

$\varepsilon_{it}/u_{i}\sim N(0,\theta)$ $u_{i}\sim N(0,\varphi)$

$${{y_{it}=\beta_{0}+\beta_{1}{env}_{it}+\beta_{2}{age}_{i}+\beta_{3}gender}_{i}+\beta}_{4}{occup}_{i}+\beta_{5}{mh\_baseline}_{i}+\beta_{6}{env}_{it}*{impulse}_{i}+\varepsilon_{it}+u_{i}$$

where ~ means ‘is distributed as’. On the continuous scale, the total score of mental well –being $y_{it}$is a linear combination of the following terms. A constant term $\beta_{0}$,a random intercept $u_{i}$which represents the between subject error term and $\varepsilon_{it}$ represents the within subject error term. In addition,${env}_{it}$ represents the self-reported environmental features of participant *i* at assessment *t*, ${age}_{i}$ represents the age of participant *i,*${gender}_{i}$ represents the gender of participant *i,* ${occup}_{i}$ represents the occupational status of participant *i*$,$ ${mh\_baseline}_{i}$ represents mental wellbeing of participant *i* over previous two weeks from the start of using the Urban Mind tool, and ${env}_{i}*{impulse}_{i}$ represents an interaction term of self-reported environmental features with trait impulsivity of participant *i*, and $\beta_{1},\beta_{2},\beta_{3}, \beta_{4},\beta_{5},\beta_{6}$are the corresponding respective fixed effects terms$.$

***Table S1. Pearson and Goodman & Kruskal’s gamma correlation coefficients between covariates in our statistical models. *Pearson correlation coefficient; ** Goodman & Kruskal's gamma correlation coefficient.***

|  | Mental wellbeing over previous two weeks | Age | Gender | Occupational status |
| --- | --- | --- | --- | --- |
| Mental wellbeing over the previous two weeks | 1 |  |  |  |
| Age | -0.04* | 1 |  |  |
| Gender | 0.11** | 0.17** | 1 |  |
| Occupational status | -0.17** | -0.13** | 0.02** | 1 |
| Are you indoors or outdoors? | 0.04** | 0.09** | 0.06** | -0.07** |
| Can you see trees? | 0.06** | 0.03** | -0.006** | -0.002** |
| Can you hear birds singing? | -0007** | 0.09** | 0.013** | 0.06** |
| Can you see or hear water? | 0.09** | -0.13** | 0.04** | -0.003** |
| Can you see the sky? | 0.03** | 0.03** | -0.05** | 0.10** |
| Do you feel in contact with nature? | 0.008** | -0.05** | 0.10** | 0.03** |

***Table S2. Associations between momentary mental wellbeing score in relation to self-reported environmental features adjusted for age, gender, occupational status and mental wellbeing over the previous two weeks for different thresholds of completed assessments (> 33%, >50%, and >66%). Mean Difference (MD) and 95% Confidence Intervals (CI) represent a mean difference in momentary mental wellbeing score per category increase.***

|  | MD  (95% CI) | *p-value* | MD  (95% CI) | *p-value* | MD  (95% CI) | *p-value* |
| --- | --- | --- | --- | --- | --- | --- |
|  |  |  |  |  |  |  |

|  | N=108 (33%) |  | N=64 (50%) |  | N=25 (66%) |  |
| --- | --- | --- | --- | --- | --- | --- |
| Are you indoors or outdoors? | 2.90  (1.48, 4.33) | <0.001 | 2.41  (1.43, 3.39) | <0.001 | 2.31  (1.52, 3.11) | <0.001 |
| Can you see trees? | 1.31  (0.16, 2.46) | 0.025 | 1.82  (1.06, 2.57) | <0.001 | 1.78  (1.14, 2.42) | <0.001 |
| Can you hear birds singing? | 2.49  (-0.31, 5.29) | 0.081 | 3.71  (1.75,5.68) | <0.001 | 3.82  (2.21, 5.43) | <0.001 |
| Can you see or hear water? | 3.24  (0.14, 6.33) | 0.040 | 1.20  (-1.07,3.48) | 0.30 | 1.33  (-0.55, 3.21) | 0.166 |
| Can you see the sky? | 0.94  (-0.30,2.19) | 0.136 | 1.63  (0.79,2.46) | <0.001 | 1.42  (0.69,2.14) | <0.001 |
| Do you feel in contact with nature? | 1.28  (-1.31,3.86) | 0.333 | 3.51  (1.62,5.41) | <0.001 | 3.77  (2.20, 5.34) | <0.001 |

|  |
| --- |

***Table S3. Associations of momentary mental wellbeing score in relation to self-reported environmental characteristics adjusted for age, gender, occupation and mental wellbeing over the previous two weeks with the use of Multiple Imputation with Chained Equations*** (***MICE) procedure. Mean Difference (MD) and 95% Confidence Intervals (CI) represent a mean difference in momentary mental wellbeing score per category increase.***

|  |  | MD (95% CI) | *p-value* |
| --- | --- | --- | --- |
| Are you indoors or outdoors? | L0^±^ | 2.45 (0.86,4.04) | 0.003 |
|  | L1^±^ | 1.41 (-0.18,3.00) | 0.083 |
|  | L2^±^ | -0.85 (-2.42,0.71) | 0.286 |
|  | L3 | 0.41 (-1.24,2.06) | 0.630 |
| Can you see trees? | L0 | 1.26 (-0.01,2.53) | 0.052 |
|  | L1 | 1.62 (0.31,2.92) | 0.015 |
|  | L2 | -0.76 (-2.08,0.56) | 0.261 |
|  | L3 | -0.31 (-1.70,1.07) | 0.654 |
| Can you hear birds singing? | L0 | 4.02 (1.39,6.66) | 0.008 |
|  | L1 | 1.47 (-0.99,3.93) | 0.206 |
|  | L2 | 1.18 (-0.54,2.89) | 0.166 |
|  | L3 | 0.86 (-0.42,2.15) | 0.187 |
| Can you see or hear water? | L0 | 1.72 (-1.34,4.78) | 0.229 |
|  | L1 | 0.86 (-0.81,2.53) | 0.306 |
|  | L2 | -0.12 (-1.97,1.74) | 0.896 |
|  | L3 | 0.37 (-1.53,2.27) | 0.691 |
| Can you see the sky? | L0 | 1.06 (-0.47, 2.59) | 0.170 |
|  | L1 | 1.46 (0.13,2.80) | 0.031 |
|  | L2 | -0.38 (-1.68,0.92) | 0.570 |
|  | L3 | -0.85 (-2.32,0.63) | 0.258 |
| Do you feel in contact with nature? | L0 | 3.49 (1.75,5.23) | 0.001 |
|  | L1 | 1.64 (-0.52,3.79) | 0.120 |
|  | L2 | 1.70 (0.31,3.10) | 0.018 |
|  | L3 | 0.53 (-2.34,3.41) | 0.671 |

^±^ As the interval between successive ecological momentary assessments was 2 hours and 25 minutes on average, one-lagged effects (L1) would indicate that the impact of a self-reported environmental feature of interest was still evident after 2 hours and 25 minutes; two-lagged effects (L2) would indicate that the impact of a feature of interest was still evident after 4 hours and 50 minutes; and three-lagged effects (L3) would indicate that the impact of a feature of interest was still evident after 7 hours and 15 minutes.

***Table S4. Associations between momentary mental wellbeing and the interaction between trait impulsivity score and self-reported environmental features, adjusted for age, gender, occupation and mental wellbeing over the previous two weeks for > 50% completed assessments. Mean Difference (MD) and 95% Confidence Intervals (CI) represent a mean difference in momentary mental wellbeing score per category increase.***

|  | MD  (95% CI) | *P for interaction term* |
| --- | --- | --- |
| Are you indoors or outdoors? | -0.02 (-0.31, -0.12) | .037 |
| Can you see trees? | -0.24 (-0.45, -0.03) | .026 |
| Can you hear birds singing? | 0.72 (0.11, -1.32) | .021 |
| Can you see the sky? | -0.09 (-0.81, -0.63) | .805 |
| Can you see or hear water | -0.40 (-0.63, -0.17) | .001 |
| Do you feel in contact with nature? | 0.45 (0.22, -0.92) | 0.02 |

**Scale S1. Warwick-Edinburgh Mental Well-being Scale Used for Baseline Assessment of Mental Wellbeing.**

***Next are some statements about feelings and thoughts. Please indicate your experience of each over the last 2 weeks by tapping on the relevant number/answer.***

1. I feel optimistic about the future.
2. I feel useful.
3. I feel relaxed.
4. I feel interested in other people.
5. I feel I have energy to spare.
6. I deal with problems well.
7. I think clearly.
8. I feel good about myself.
9. I feel close to other people.
10. I feel confident.
11. I am able to make up my own mind about things.
12. I feel loved.
13. I am interested in new things.
14. I feel cheerful.

***Responses and associated scores:***

I very much disagree (score: 1)

I slightly disagree (score: 2)

Not sure (score: 3)

I slightly agree (score: 4)

I very much agree (score: 5)

***Total Scoring:***

Total score is computed by adding the scores for individual items.

**Scale S2. Trait Rash Impulsivity Scale Used for Baseline Assessment of Trait Impulsivity.**

***Please read each statement and indicate to what extent it applies to you by tapping the appropriate number/answer. Answer quickly and honestly, without spending too much time on any statement.***

1. I am focused, seeing things through to the end.
2. I plan work tasks and activities in my free time carefully.
3. I plan events and activities well ahead of time.
4. I think carefully before doing and saying things.
5. I find it easy to exercise self-control.
6. I encounter problems because I do things without stopping to think.
7. I become involved with things that I later wish I could get out of.
8. I tend to jump from one interest to another.
9. I tend to act ‘on impulse’.

***Responses and associated scoring:***

“Rarely / Never” equals a score of 1 for questions 6, 7, 8 & 9; and a score of 4 for questions 1, 2, 3, 4 & 5)

“Occasionally” equals a score of 2 for questions 6, 7, 8 & 9; and a score of 3 for questions 1, 2, 3, 4 & 5)

“Often” equals a score of 3 for questions 6, 7, 8 & 9; and a score of 2 for questions 1, 2, 3, 4 & 5)

“Almost / Always” equals a score of 4 for questions 6, 7, 8 & 9; and a score of 1 for questions 1, 2, 3, 4 & 5)

***Total Scoring:***

Total score is computed by adding the scores for individual items.

**Scale S3. Adapted version of the Warwick-Edinburgh Mental Well-being Scale Used for Ecological Momentary Assessment of Mental Wellbeing.**

***Next are some statements about feelings and thoughts. Please indicate your agreement with each of them by tapping on the relevant number/answer.***

1. Right now I feel optimistic about the future.
2. Right now I feel useful.
3. Right now I feel relaxed.
4. Right now I feel interested in other people.
5. Right now I feel I have energy to spare.
6. Right now I deal with problems well.
7. Right now I think clearly.
8. Right now I feel good about myself.
9. Right now I feel close to other people.
10. Right now I feel confident.
11. Right now I am able to make up my own mind about things.
12. Right now I feel loved.
13. Right now I am interested in new things.
14. Right now I feel cheerful.

***Possible responses and associated scores:***

I very much disagree (score: 1)

I slightly disagree (score: 2)

Not sure (score: 3)

I slightly agree (score: 4)

I very much agree (score: 5)

***Total Scoring:***

Total score is computed by adding the scores for individual items.
